# A Scale-Free, Fully Connected Global Transition Network Underlies Known Microbiome Diversity

**DOI:** 10.1128/mSystems.00394-21

**Published:** 2021-07-13

**Authors:** Gongchao Jing, Yufeng Zhang, Lu Liu, Zengbin Wang, Zheng Sun, Rob Knight, Xiaoquan Su, Jian Xu

**Affiliations:** a Single-Cell Center, CAS Key Laboratory of Biofuels and Shandong Key Laboratory of Energy Genetics, Qingdao Institute of BioEnergy and Bioprocess Technology, Chinese Academy of Sciences, Qingdao, Shandong, China; b College of Computer Science and Technology, Qingdao University, Qingdao, Shandong, China; c University of California, San Diego, California, USA; d University of Chinese Academy of Sciences, Beijing, China; University of Massachusetts Medical School

**Keywords:** microbiome transition, scale-free, network, data mining, beta diversity

## Abstract

Microbiomes are inherently linked by their structural similarity, yet the global features of such similarity are not clear. Here, we propose as a solution a search-based microbiome transition network. By traversing a composition-similarity-based network of 177,022 microbiomes, we show that although the compositions are distinct by habitat, each microbiome is on-average only seven neighbors from any other microbiome on Earth, indicating the inherent homology of microbiomes at the global scale. This network is scale-free, suggesting a high degree of stability and robustness in microbiome transition. By tracking the minimum spanning tree in this network, a global roadmap of microbiome dispersal was derived that tracks the potential paths of formulating and propagating microbiome diversity. Such search-based global microbiome networks, reconstructed within hours on just one computing node, provide a readily expanded reference for tracing the origin and evolution of existing or new microbiomes.

**IMPORTANCE** It remains unclear whether and how compositional changes at the "community to community" level among microbiomes are linked to the origin and evolution of global microbiome diversity. Here we propose a microbiome transition model and a network-based analysis framework to describe and simulate the variation and dispersal of the global microbial beta-diversity across multiple habitats. The traversal of a transition network with 177,022 samples shows the inherent homology of microbiome at the global scale. Then a global roadmap of microbiome dispersal derived from the network tracks the potential paths of formulating and propagating microbiome diversity. Such search-based microbiome network provides a readily expanded reference for tracing the origin and evolution of existing or new microbiomes at the global scale.

## INTRODUCTION

Microbiome composition, a fundamental feature of all microbiota in nature, is shaped by a plethora of environmental factors, such as habitats, geographic locations, temperature, oxygen level, and even day length (1, 2). However, it remains unclear whether and how compositional changes at the “community-to-community” level among microbiomes are linked to the origin and evolution of global microbiome diversity (3–5). For example, did microbiomes from different environments emerge and develop separately, or did the global microbiome start homologically and then spread to other habitats with compositional dispersal and dynamics (6)? Over recent years, a large number of microbiome samples (e.g., Human Microbiome Project (7), Earth Microbiome Project (1), Tara Ocean Project (8), etc.), mainly in the form of 16S rRNA amplicons, have been produced and accumulated (4); however, the ability to cluster and model microbiomes at the global scale has been hindered by the enormous volume and sheer complexity of such data (e.g., a distance matrix of 100,000 microbiomes contains ∼5 × 10^9^ elements).

## RESULTS

### Microbiome transition model and search-based network.

**FIG 1:**
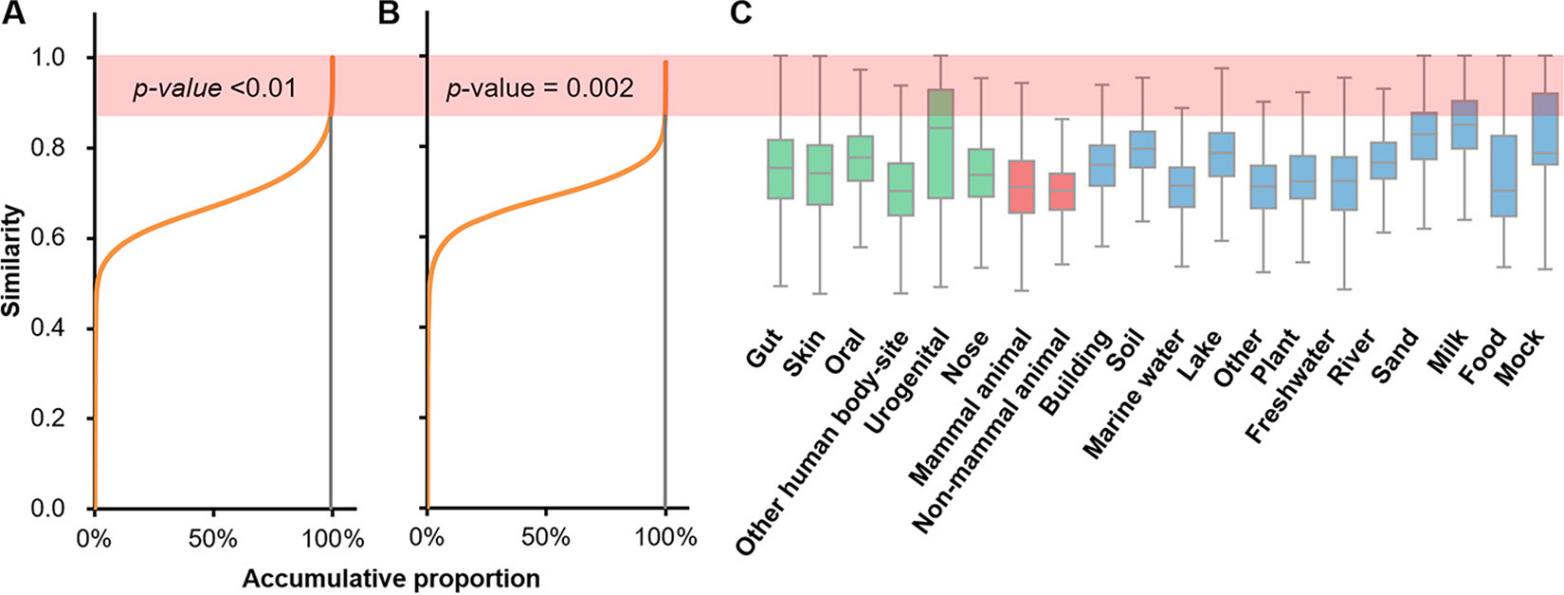
Distribution of pairwise similarity in *n *= 177,022 microbiome samples. (A) A *P* value of <0.01 for significant similarity values in the permutation determined the threshold of 0.868 (under the shadow) for putative direct transition. (B) The threshold has *P* value = 0.0022 among between-habitat similarity distribution. (C) The threshold is higher than the upper boundary of within-habitat similarities for most habitats. The three panels use the same *y* axis. *P* values are calculated by permutation test.

Here, we describe the compositional dynamics and variation among microbial communities by a *microbiome transition* model. In this model, a microbial community is essentially a combination of microorganisms and the structure of a community can be modified to another form by adding and/or removing species by compositional dispersal or fusion (9, 10) (Fig. S1 in the supplemental material). Theoretically, higher similarity between two communities indicates higher probability for such microbiome transition, since fewer compositional exchanges are needed; however, it is not clear what level of similarity may indicate such microbial transition with reasonable confidence. Based on a pairwise full permutation of similarity calculation among all microbiomes from the Microbiome Search Engine (MSE) database (MSE is a microbiome database platform for searching query microbiomes against the global metagenome data space based on the whole-community-level similarity [11]; it contains 177,022 samples in total) (refer to the “Microbiome sample collection” section for details) using the Meta-Storms algorithm (12, 13) (Table 1), we consider that “direct transition” possibly exists between sample pairs with significant similarities that cause permutation *P* values of <0.01 (equation 1; Fig. 1). As the result, we define the Meta-Storms similarity of 0.868 as the threshold for direct transition between microbiomes. By further analyzing the pairwise similarity in each habitat, we found that the threshold similarity of 0.868 is significantly high in the between-habitat similarity distribution (*P* value = 0.0022) (Fig. 1B); moreover, it is higher than the upper boundary of most within-habitats similarities (17 of 20; Fig. 1C). Thus, the similarity threshold of 0.868 is sufficiently stringent for defining microbiome transition among the ecosystems.

**TABLE 1:** Distribution of samples among the habitats

Sample type	Habitat	Source	No. of samples
Human-associated	Gut	Feces, etc.	51,706
	Skin	Hand, arm, head, leg, etc.	19,455
	Oral	Tongue, saliva, plaque, etc.	10,896
	Other human body-site	Hair, lung, blood, eye, etc.	3,018
	Urogenital	Vagina, urine, etc.	1,204
	Nose	Nostril	489
Animal-associated	Mammal animal	Mouse, rabbit, dog, deer, etc.	29,918
	Nonmammal animal	Sponge, fish, insect, etc.	11,172
Environmental	Building	Indoor environment, etc.	11,248
	Soil	Grass cover, cropland, soil sediment, etc.	10,507
	Marine water	Sea water	6,090
	Lake	Lake water, lake sediment, etc.	4,234
	Plant	Plant rhizosphere, plant surface, etc.	3,456
	Freshwater	Blank control, tap water, etc.	3,112
	River	River water, river sediment, etc.	2,248
	Milk	Tanker milk, blended solo milk, etc.	1,636
	Sand	Beach, desert, sand sediment, etc.	968
	Food	Food surface, etc.	780
Other	Other	Other environment	4,074
	Mock	Mock microbiome	811
Total			177,022

10.1128/mSystems.00394-21.1 FIG S1 Microbiome transition model. A microbial community is a combination of microorganisms and the composition of one community could be modified to another form by increasing and/or decreasing the amount of its species, or by adding and/or removing species from the community. Download FIG S1, TIF file, 0.2 MB. Copyright © 2021 Jing et al. 2021 Jing et al. https://creativecommons.org/licenses/by/4.0/ This content is distributed under the terms of the Creative Commons Attribution 4.0 International license.

Then, for each of the input 177,022 microbiomes, we applied MSE to search against all other samples and find the top matches with similarity higher than 0.868. Based on the search results, we constructed a transition network in which each node is a microbiome and each edge represents a direct transition (equation 2; Fig. S2). Collectively, the network consists of 177,022 nodes (samples) and 11,175,742 edges (each called a direct transition). Notably, a pair of samples with low similarity can be connected via multiple edges (i.e., via a series of direct transitions across intermediate transfer samples), and such a sample pair is termed an “indirect transition” (equation 3).

10.1128/mSystems.00394-21.2 FIG S2 Construction of search-based microbiome transition network. (A) For a query sample *a*, we search it against all other samples and connect it with the matched samples *b*, *c*, and *d* that have similarity higher than the threshold of direct transition. (B) We then iterate this procedure to sample *b* and connect it with *e*, *f*, and *g*. (C) By iterating the search through all samples, we construct a global transition network. Download FIG S2, TIF file, 0.4 MB. Copyright © 2021 Jing et al. 2021 Jing et al. https://creativecommons.org/licenses/by/4.0/ This content is distributed under the terms of the Creative Commons Attribution 4.0 International license.

### The transition network predicts microbiome habitat at a global scale.

**FIG 2:**
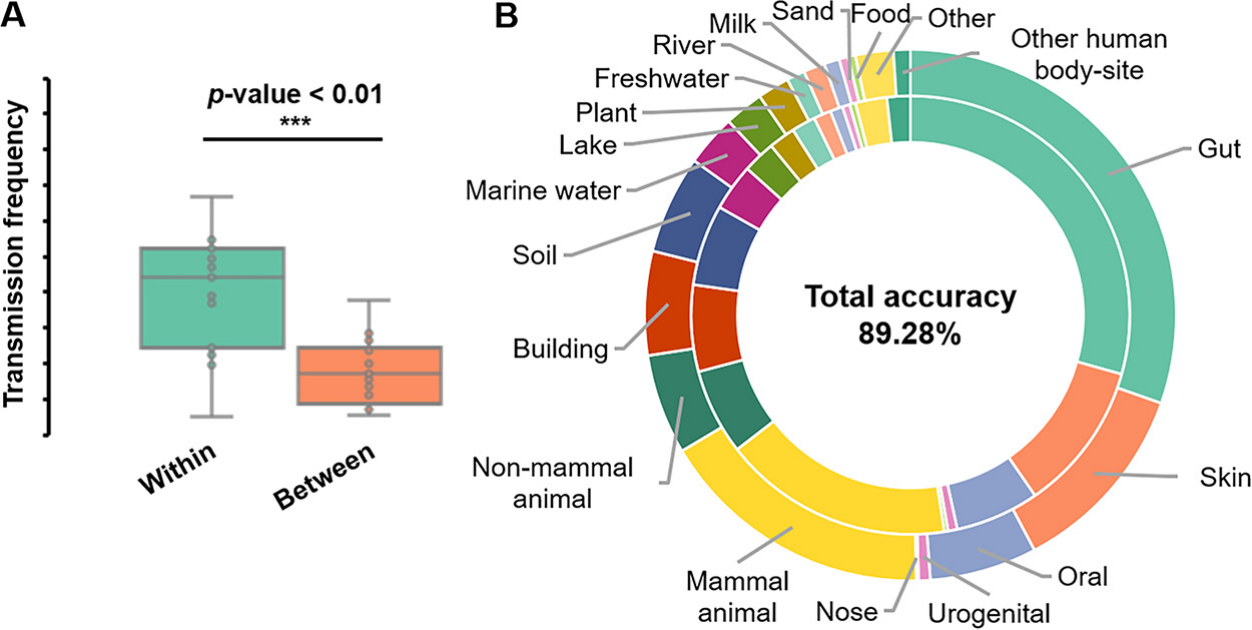
Global microbiome network predicts the microbiome habitat. (A) Frequency of within-habitat direct transition is significantly higher than that of between-habitat. *P* value is calculated by a two-sided *t* test. (B) The habitat of 89.28% of samples is correctly predicted by the microbiome network. The inner ring represents the proportion of real habitats and the outer ring is the proportion of predicted habitats.

At the global scale, it is still not clear whether (and to what degree) similarity in microbiome structure implies similarity in ecosystem features (8, 14). To quantitatively tackle this question, we compared the direct transition frequency between within-habitat (transitions of sample pairs from the same habitat) and between-habitat (transitions of sample pairs between two different habitats) cases in the transition network. For each habitat, the direct transition frequency is calculated by the average number of direct transitions per sample in this habitat. Notably, the direct transition exists more frequently between samples in the same habitat (Fig. 2A; two-tailed paired *t* test, *P* value < 0.01). Thus, the source environment of microbiomes dominates the microbial composition. We next used the transition network to predict the habitat (mock samples were not included) of each sample by its top neighbors (see the Materials and Methods). Via leave-one-out cross validation (LOOCV), 89.28% of samples were correctly assigned by their original habitats (Fig. 2B; Table 2). Therefore, at a global scale, microbiome structure is strongly correlated with their environmental features.

**TABLE 2:** Prediction of habitat based on the microbiome network

Habitat	No. of samples	No. of correctly predicted samples	% Accuracy	Within-habitat transition frequency	Between-habitat transition frequency
Gut	51,706	50,431	97.53	66.89	5.40
Skin	19,455	17,464	89.77	52.11	17.82
Oral	10,896	10,070	92.42	55.10	10.98
Other human body-site	3,018	1,777	58.88	24.64	37.92
Urogenital	1,204	1,046	86.88	44.16	17.29
Nose	489	91	18.61	5.00	55.29
Mammal animal	29,918	28,010	93.62	50.67	8.87
Nonmammal animal	11,172	8,077	72.30	19.81	19.95
Building	11,248	8,942	79.50	40.24	24.54
Soil	10,507	9,978	94.97	54.49	7.28
Marine water	6,090	3,960	65.02	24.29	17.77
Lake	4,234	3,983	94.07	49.64	7.35
Plant	3,456	3,127	90.48	46.91	15.16
Freshwater	3,112	1,671	53.70	22.51	28.66
River	2,248	2,011	89.46	36.84	15.55
Milk	1,636	1,565	95.66	55.21	8.23
Sand	968	864	89.26	44.72	23.54
Food	780	677	86.79	39.01	26.57
Other	4,074	3,573	87.70	38.88	13.64

“Mismatches,” i.e., microbiomes that were assigned to an incorrect habitat as predicted by the transition network, represent 10.72% (18,894 of 176,211) of all samples. Such mismatches are interesting as they can be caused by, and thus potentially indicate, frequent contact and interchanges of microbiota among the habitats. Those among human body sites are the most frequently observed mismatches (1.86% of all samples, same as below) caused by the daily contact and exchange of microbial composition (15). Matches between nonmammal animal (sponge) and marine water are the second most frequent mismatches (1.76%). Mismatches across “human skin,” “animal (pet),” and “building (indoor environment)” represent 1.68% of samples, likely due to the sharing of the indoor environment (where the microbiome was largely sourced from humans) (5, 16). Moreover, 0.81% of the mismatches are between human-gut and mammal-animal-gut, which can be explained by the close phylogenetic relationship between human and other mammals and the coevolution of mammals and their gut microbiota (17). Furthermore, mismatches are also observed (0.14%) where lake water samples are predicted as river water (note that this is the source stream of lake), or vice versa. Therefore, although the microbiome structure at the global scale is mainly shaped by their habitat, microbiome structure can be altered by, and thus reflect, the contact and exchange of microbiota from different environments.

### Microbiomes are connected globally by the transition network.

The beta diversity of global microbiomes may have evolved via two scenarios: (i) “polyphyly,” where microbiomes from different environments were generated and developed separately (Fig. S3A), or (ii) “monophyly,” in which microbiomes started homologically and then were dispersed to other habitats (e.g., via compositional transition, exchange, or fusion) (Fig. S3B). To distinguish between the two scenarios, we used the transitive closure algorithm to examine the connectivity of this transition network (see the Materials and Methods). A closure is a set of nodes (microbiomes) in which each microbiome can traverse to any other one by direct or indirect transitions (with finite steps). Hence, being in a closure implies likelihood of transformation among samples via compositional exchange. Traversing all nodes in the network via the transitive closure algorithm revealed that 98.31% of samples (174,032 of 177,022) can be clustered into a single closure (also named as the “main closure”). Under a condition that microbiota composition is distinct by habitat for 89.28% of all samples (Fig. 2B), such high connectivity suggests that the likelihood of polyphyly should be very low (probability < 1.5e−05; estimated by equation 5) and supports the monophyletic origin of global microbiomes and the formation of new microbiomes via such transitions (Fig. S3B). Notably, 1.69% (2,990 of 177,022) of samples are still not included in the main closure, and they were mostly due to statistical inaccuracy (1.47% exhibit a similarity level that is only slightly below the threshold for being recruited into the main closure; *P* value between 0.01 and 0.05) or curation errors (e.g., 0.16% are labeled as microbiome but actually pure-cultures or 18S/ITS amplicon samples). Therefore, the monophyly hypothesis best explains the origin and evolution of present-day microbiome structures.

10.1128/mSystems.00394-21.3 FIG S3 Two scenarios of the global microbiome generation. (A) Microbiomes of different environments were generated and developed separately. (B) The global microbiome started from the same ancestors and then spread to other habitats with compositional transition and exchange. Download FIG S3, TIF file, 0.3 MB. Copyright © 2021 Jing et al. 2021 Jing et al. https://creativecommons.org/licenses/by/4.0/ This content is distributed under the terms of the Creative Commons Attribution 4.0 International license.

To size the global microbiome network, we computed the pairwise shortest transition steps of all sample pairs in the main closure using the Dijkstra algorithm (18) (see the Materials and Methods). Interestingly, like the “small world” principle for social network (19), the microbiome transition network follows the “7-degree of separation” pattern (Fig. S4A). Specifically, any two microbiota in the main closure, even if they were sampled from different habitats and exhibit low similarity, can traverse from one to the other with only seven direct transitions on average (20), and 32 such steps at the maximum (i.e., the network diameter) (Fig. S4B). Such a pattern underscores the high connectivity and, thus, the surprisingly close interaction among microbiomes from diverse habitats at the planetary scale.

10.1128/mSystems.00394-21.4 FIG S4 The “small world” principle of the microbiome transition network. (A) Any two microbiomes in the main closure could be transitioned to one another by a 7-step transition on average. (B) Distribution of the pairwise shortest transition steps of all sample pairs. Download FIG S4, TIF file, 0.4 MB. Copyright © 2021 Jing et al. 2021 Jing et al. https://creativecommons.org/licenses/by/4.0/ This content is distributed under the terms of the Creative Commons Attribution 4.0 International license.

### Global microbiome network is scale-free and the connectivity is robust.

**FIG 3:**
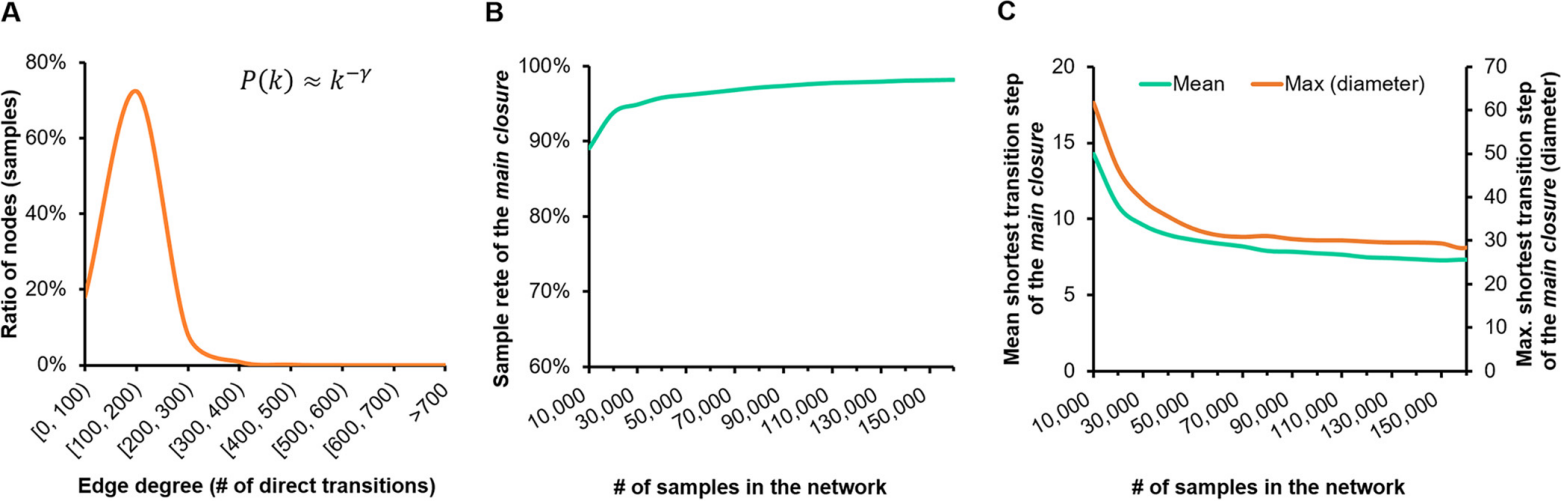
Robustness of the global microbiome network. (A) Node degree (number of linked neighbors) of the network follows the Poisson distribution, suggesting the network is scale-free. (B) The effect of random node removal on the main closure in as function of sample rate. (C) The mean shortest transition step and maximum transition step (diameter).

Notably, in this global transition network, for each node, its edge degree *k* (number of direct transition neighbors) follows a Poisson distribution (Fig. 3A), where Pearson *r* = −0.836 between *log*(*P*[*k*]) and *log*(*k*), i.e., $P(k)\approx k^{\left(-\gamma \right)}$), suggesting that the network is scale-free (21, 22). One key feature of a scale-free network is the stability of topology, i.e., robustness to node removal from the network. To test the robustness, we removed different numbers of randomly selected samples and their associated edges, then assessed both size and leftover sample rate of the main closure in the residual network (percentage of residual nodes ranged from 5% to 95%; each such removal procedure was repeated for 10 times) (see the Materials and Methods). When the number of nodes in the network reaches 80,000 (45% of total nodes), the connectivity rate curve of the main closure already exhibits a flat trend with 97.19% samples (98.31% prior to sample removal) (Fig. 3B) and, moreover, the mean transition steps and maximum transition steps (diameter) converge to 8 and 33, respectively (which were 7 and 32 before sample removal) (Fig. 3C). Thus, these parameters are quite stable and not dependent on the increase of total sample number in the network. These findings suggest the robustness of microbiome diversity and similarity patterns among ecosystems at the global scale.

### Microbiome transition roadmap simulates the development of global microbial diversity among multiple ecosystems.

**FIG 4:**
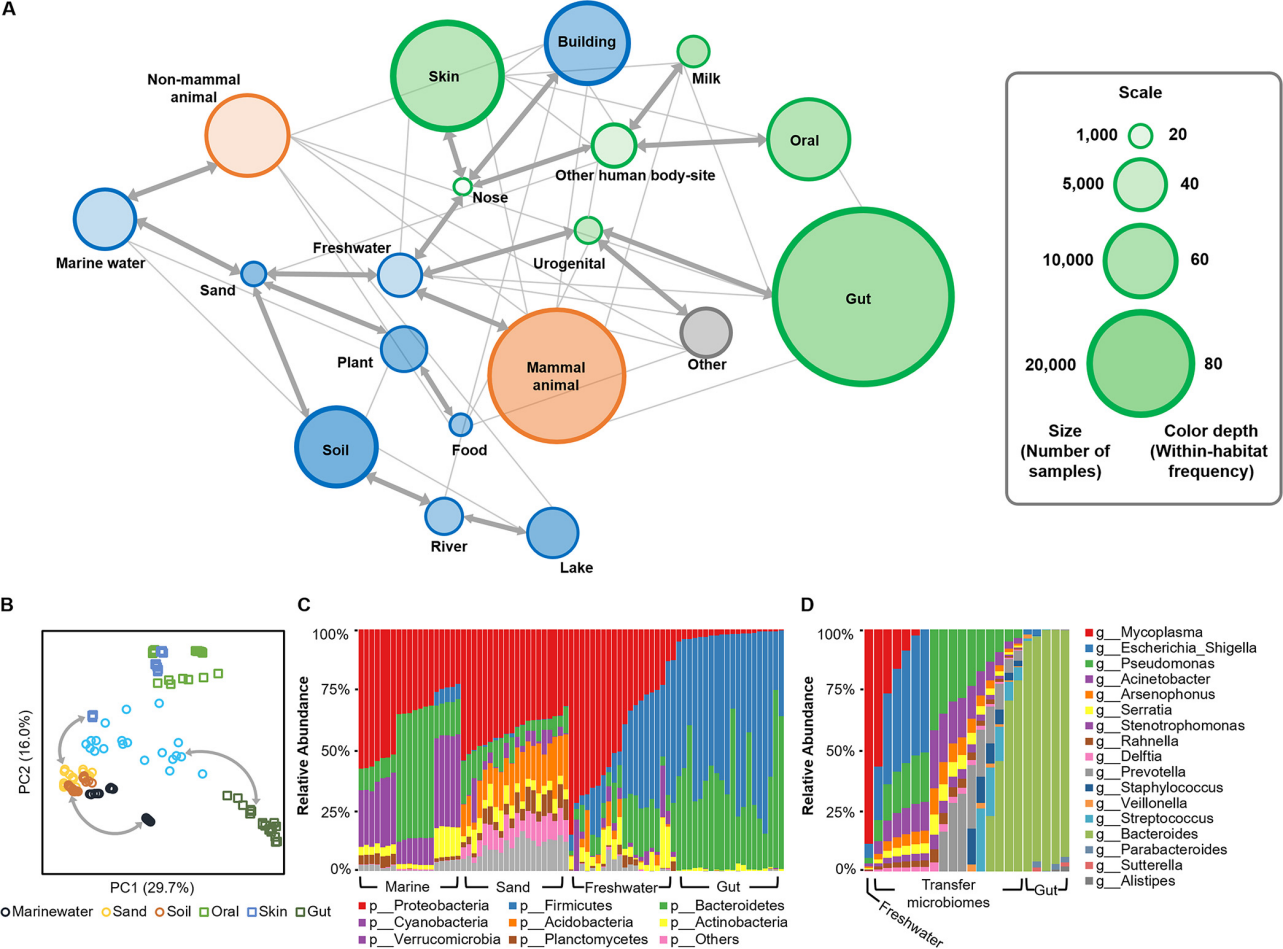
Roadmap of the global microbiome transition among habitats. (A) Bold lines are the roadmap that represents the maximum overall similarity, in which arrows indicate the transitions are bidirectional. The number of samples in each habitat is scaled by the node size, and the within-habitat transition frequency is represented by the node color depth (compared to the rim). Thin lines show the high frequent transitions between habitats. (B) Principle-coordinate analysis (PCoA) parsed from a subset of 140 microbiomes demonstrates the roadmap by the equivalent topology. (C) The phylum-level compositional shift of a microbiome transition route for marine to gut environment. (D) The genus-level compositional shift of a transition case from a freshwater microbiome to gut samples.

As microbiome compositions are dominantly determined by their habitats, the full connectivity of global microbiomes in the network suggests the ability to reconstruct how the microbial diversity spreads among different habitats at a macroscopic scale. This “microbial dispersal” roadmap can be simulated by a subnetwork that (i) covers and links all samples, and (ii) consists of deterministic finite transition steps without cyclic or redundant routes. Thus, we derived such a roadmap (Fig. 4A) by parsing the minimum spanning tree (MST) of the main closure using the Kruskal algorithm (23) (see the Materials and Methods). As the global optimum with the highest overall transition probability (similarity), the MST maximally captures the transition pattern of worldwide microbial diversity among all the 19 habitats (with the “mock” samples excluded). For example, marine microbiomes most probably exchange with two other environments, where one is sand, which is geographically close to the shore, while the other is nonmammal animals such as fishes. These observations also suggest that sand and freshwater microbiomes are the “gateways” to soil, plants, and human-associated habitats such as gut, oral, skin, and the human living environments.

This roadmap is verified by the isomorphic pattern of principal-coordinate analysis (PCoA) derived from a subset of 140 samples randomly selected from six habitats (Fig. 4B). Moreover, we employed a marine-gut route, which represents one of the longest transitions in this subset, to illustrate the high-resolution transition procedures (based on phylum-level compositional variations; urogenital and gut microbiomes were combined as they are very close in the global scale) (Fig. 4C). Zooming in on this marine-gut route revealed a series of structure shifts that transform a freshwater microbiome to gut samples. Starting from an actual freshwater sample (24), in each step, organisms enriched in freshwater (25) (e.g., *Mycoplasma* and Escherichia) were removed/reduced, and organisms abundant in gut (15) (e.g., *Bacteroides* and *Parabacteroides*) were added/increased. Although a single step might have caused just slight modification on the microbiome structure, after several iterations this sample can be smoothly transited to gut microbiomes (26) in the network, via a series of transfer samples (Fig. 4D).

### Microbiome transition over time and across geography.

**FIG 5:**
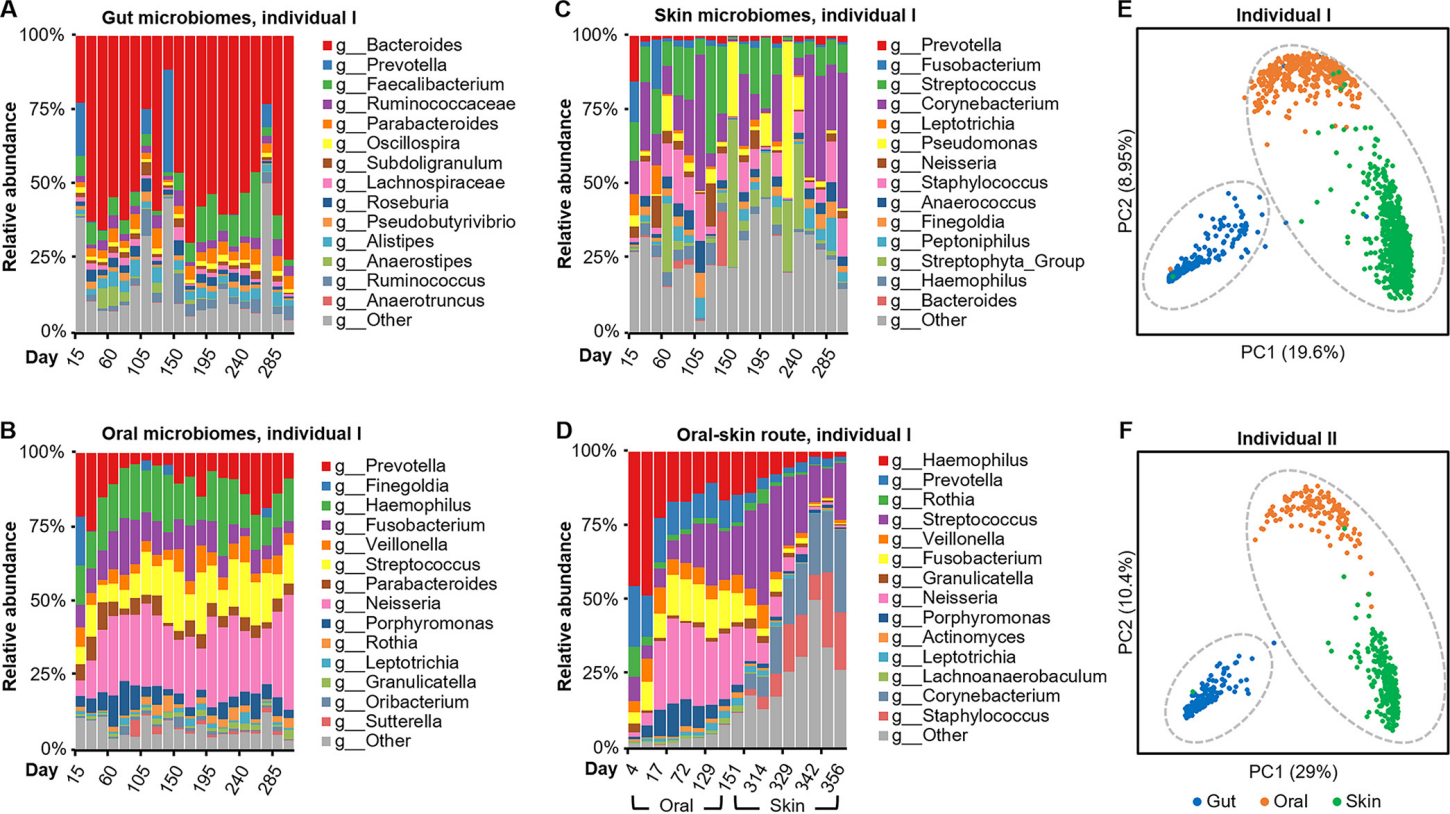
Transition of the human microbiome across time and body sites. (A to C) The within-habitat microbiome transition of gut (A), oral (B), and skin (C) of individual I across 396 time points. (D) The oral-skin microbiome transition of individual I across time. Only selected samples are shown. The transition patterns of individual II are shown in Fig. S5 in the supplemental material. (E and F) PCoA of the two individuals’ time-series microbiomes: skin and oral microbiomes are linked in a closure by direct transition (highlighted by gray dotted line) and gut samples form another closure.

To test the feasibility of modeling microbial dynamics by the global microbiome transition network, we used a longitudinal cohort to describe the transition of human microbiomes across time. In this data set, 1,963 samples were collected from three body sites (gut, oral cavity, and skin) of two individuals (I, male; II, female) over 396 time points (27). Our search-based network analysis revealed that the microbiome composition of each body site exhibits significant variations across time (Fig. 5A to C; Fig. S5), while skin and oral microbiomes were clustered into the same closure by direct transition (Fig. 5D to F). These suggest that microbiome transition is ongoing within each site and between the skin and oral sites across different time points. In addition, for both hosts, gut samples were “isolated” from the skin-oral closure, consistent with the global microbiome transition map (Fig. 4A), where gut microbiomes are in a distinct route from skin and oral ones. Therefore, although the oral cavity and gut are both of the digestive tract and microbial translocation from oral to gut can occur (28), the oral microbiota have more likely been derived from (or more prominently shaped by) the skin microbiota (and vice versa) than the gut microbiota. This seemingly counterintuitive finding can actually be lent support by the sharing of a more aerobic and less acidic environment by the skin and oral cavity (pH and oxygen level are known to have large effect size in microbiome structure) (1).

10.1128/mSystems.00394-21.5 FIG S5 Transitions of human microbiomes across time and body sites. The within-habitat microbiome transitions of gut (A), oral (B), and skin (C) of individual II across 396 time points. Download FIG S5, TIF file, 1.1 MB. Copyright © 2021 Jing et al. 2021 Jing et al. https://creativecommons.org/licenses/by/4.0/ This content is distributed under the terms of the Creative Commons Attribution 4.0 International license.

**FIG 6:**
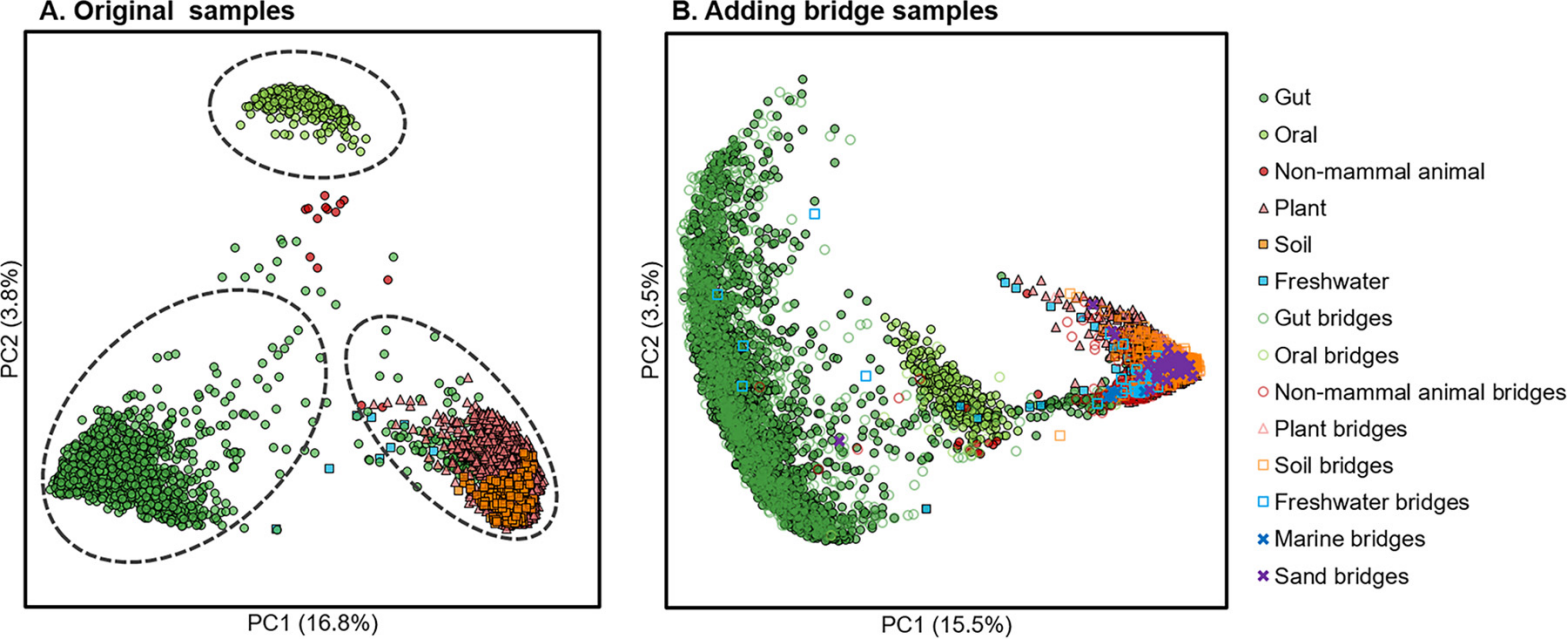
Microbiome transition across habitats and geographical locations. (A) The 3,850 samples from six habitats are included in three isolated transition closures, of which the sample proportions are 43.22%, 7.90%, and 45.53%, respectively. (B) After adding an extra 1,635 bridge samples from the MSE database, the three closures merged by direct transition into a single closure, which contains 97.74% of the samples.

On the other hand, to validate the connectivity of microbiomes from varied geographical locations, we constructed a search-based network by a single data set (29) that contains 3,850 samples collected from six habitats (human gut, human oral, nonmammal animal, plant, soil, and freshwater) and locations in North America (Urbana, IL; Columbia, MO; Aurora, CO; Ithaca, NY; and Lansing, MI, etc.). With this data set, the authors concluded there was no overlap of abundant bacterial taxa between the microbial communities from human gut and plant roots (29). Consistent with this conclusion, the network-based analysis based solely on this data set found that samples were distributed into three isolated closures of direct transitions (Fig. 6A). However, once an extra 1,635 samples from the MSE database that connected the different closures of this local network were added, a single closure that covers 97.74% samples and integrates the original three closures emerged, with the newly included samples serving as “transfer nodes” (i.e., samples that link two clusters in network) (equation 3 in the Materials and Methods) that provide additional indirect transitions (Fig. 6B; Materials and Methods). Notably, among such “transfer” microbiomes, most (96.89%) were from the same habitats as the original data set and others were mainly from sand and marine, which are found as the transfer nodes among nonmammal animal, plant, and soil microbiomes in our global microbiome transition roadmap (Fig. 4). This example demonstrates that although microbiomes from diverse environments and isolated geographical locations can have very distinct structures, they can still be linked within a single closure in the microbiome transition network, i.e., evolve from each other, as long as global beta-diversity is adequately surveyed and covered. These results, which directly challenge the conclusion that multiple host microbiota compositions were independently evolved (29), underscores the importance of deriving or validating “local” data sets under the context of our global microbiome network, particularly when discussing similarity (i.e., beta-diversity), interaction, or other kinds of relationship among microbiomes.

## DISCUSSION

Microbiota have been coevolving with and shaping our planet, but their origin and evolution at the global scale remain elusive, due to the lack of fossils and the methodological challenges associated with integrating and mining such large-scale high-complexity data (14). Although “species-to-species” interactions have been mapped by co-occurrence analysis on microbiomes across various habitats (30, 31, 38), efforts to globally traverse and interrogate the vast microbiome data space at the “community-to-community” level have just started (32–34). Here, we propose a microbiome transition model and a network-based analysis framework to describe and simulate the variation and dispersal of the global microbial beta-diversity across multiple habitats. Benefitting from the extremely high search speed of the Microbiome Search Engine (11), we introduced a global microbiome network with 177,022 microbiome samples that contains 11.3 billion sequences. By traversing such a network, we showed the microbiome structures are connected world-wide by significant similarity that follows the “small world” principle. This endeavor reveals the inherent homology of the global microbiome diversity and supports the monophyletic origin of all microbiomes on Earth. Further, we drew the first global microbiome transition roadmap to illustrate the potential and most likely paths that can explain the evolution process of global microbiomes.

Due to the ongoing exponential growth of microbiome sequencing data, current beta-diversity analysis approaches, which mainly rely on the *O*(*n^2^*)-complexity pairwise relations (*n* is the number of samples) such as principle-coordinate analysis (PCoA) and clustering, have become increasingly stressed or even impractical, particularly when computational resources are limited. Here, we tackled this challenge via a search-based network, which is built on the “neighbors,” i.e., those with the highest similarity for each sample; this strategy reduces the computational complexity to *O*(c*n) (*c* is constant, i.e., the number of neighbors), and thus enables deciphering the pairwise similarity for >100,000 microbiomes within 3 h on a single computing node. As a result, the global microbiome transition roadmap, which will be regularly updated as community resource, can serve as a reference for interpreting or validating those existing or future observations on intermicrobiome similarity, association, or interaction, since local microbiome data sets can be readily aligned to this global roadmap based on their shared nodes. Moreover, such a network-based analysis framework, which can be extended to shotgun metagenome data sets, provides a new perspective for tracking back or predicting microbiome evolution with fine resolution even at the global scale.

## MATERIALS AND METHODS

### Microbiome sample collection.

We used all the microbiome samples from the Microbiome Search Engine database (http://mse.ac.cn). Samples were collected from 572 studies/projects that included 20 habitats (Table S1 in the supplemental material). Operational taxonomic units (OTUs) were picked and annotated against Greengenes (35) full-length 16S rRNA gene sequences (version 13-8) on 97% similarity level by Parallel-META 3 (36) (version 3.4.4). Variation of 16S rRNA gene copy number was normalized based on the IMG/M database (37). We set a minimum sequence number of 500 and minimum 16S rRNA mapping rate of 80% for each sample to ensure high quality of the reference data sets. Finally, *n *= 177,022 samples with 11,302,841,991 mapped sequences assed the quality control and curation (Table S1).

10.1128/mSystems.00394-21.7 TABLE S1 Meta-data of the Microbiome Search Engine database. Download Table S1, XLSX file, 4.9 MB. Copyright © 2021 Jing et al. 2021 Jing et al. https://creativecommons.org/licenses/by/4.0/ This content is distributed under the terms of the Creative Commons Attribution 4.0 International license.

### Calculation of pairwise microbiome similarity matrix for definition of direction transition.

The pairwise similarity matrix of all *n *= 177,022 samples was entirely permuted (totally [*n* × *n*−1]/2 = 15,668,305,731 times) to examine the distribution of microbiome phylogeny similarity using the Meta-Storms algorithm (12, 13) in the Parallel-META 3 software package. By setting a cutoff *P* value of <0.01 in the permutation of the similarity (rank of top 1%), we got the Meta-Storms similarity 0.868 as the statistical threshold of the significant high value to define the direct transition (this threshold is also referred to as *T_d.t_*). Thus, the transition model can be described in the following form

(1) $$P\left(\mathrm{direct}\;\mathrm{transition}\left(a,b\right)\right)=\{\begin{array}{c} \mathrm{sim}\left(a,b\right),\;\mathrm{if}\;\mathrm{sim}\left(a,b\right)\geq \;T_{d.t}\\ 0,\;\mathrm{if}\;\mathrm{sim}\left(a,b\right)<T_{d.t} \end{array}$$

in which *s_i_* and *s_j_* are two arbitrary microbiomes, and *sim*(*s_i_*, *s_j_*) represents their Meta-Storms similarity.

### Search-based microbiome network.

The search-based microbiome network is built using the Microbiome Search Engine (MSE) (11). For each sample, we searched it against all other samples for the top 100 matches and connected it with the matched samples that have similarity higher than the threshold of direct transition (*T_d.t_* = 0.868). By iterating such search with all samples, we constructed a global network *G*.

G=(V={a,b,…}, E={edge(a,b),…})

(2) $$\mathrm{edge}\left(a,b\right)\to \mathrm{sim}\left(a,b\right)\geq T_{d.t}$$

In this network, one node (e.g., *a* or *b* in equation 2) is a single microbiome, and edges (e.g., *edge*[*a, b*] in equation 2) that link the nodes are direct transitions (Fig. S2). Finally, in the network there were 177,022 nodes (samples) and totally 11,175,742 edges (direct transitions). In this network, a pair of samples with low similarity can be connected by a path of multiple edges, i.e., an indirect transition:

$$\mathrm{indirect}\;\mathrm{transition}\left(a,\;b\right)\to \mathrm{sim}\left(a,b\right)<T_{d.t}$$

(3) $$\exists \;\mathrm{edge}\left(a,\;x_{1}\right),\;\mathrm{edge}\left(x_{2},x_{3}\right),\;\ldots \;\mathrm{edge}\left(x_{i-1},x_{i}\right),\;\mathrm{edge}\left(x_{i},b\right)$$

Here, *x_1_*, *x*_2_, …, *x_i_* are defined as the “transfer samples” that underlie the indirect transition from *a* to *b*.

### Prediction of habitat using microbiome network.

In the network *G* we predicted the source habitat of each microbiome by its top *n *= 10 neighbor samples and similarities. For an arbitrary microbiome sample *a* in the network, similarities to its top 10 neighbors are $S=\{s_{1},s_{2},\ldots ,s_{n}\}$, while the *n* neighbor were from *m* (1 ≤ *m *≤* n*) different habitats as $H=\{h_{1},h_{2},\ldots ,h_{m}\}$, then the probability for the predicted habitat of microbiome *a* as *h_k_* is calculated by

(4) $$P\left(a\in h_{k}\right)=\frac{\sum_{j\in h_{k}\;}\left(\left(n-j+1\right)\text{ * }s_{j}\right)\;}{\sum_{i=1}^{n}\left(\left(n-i+1\right)\text{ * }s_{i}\right)}$$

Here, *j∈h_k_* means the habitat of neighbor *j* is *h_k_* (1 ≤ *k *≤ m). Then the predicted habitat that has the highest probability *P* will be assigned to the sample *a* as the prediction results.

### Probability of global transition among all habitats in the network.

By equation 4 we calculated that at the global scale the microbiota composition is distinct by habitat, and that the probability of transition among the same habitat is 89.28% (Fig. 2B). To calculate the overall probability of connecting all habitats in the transition network, we can start from connecting arbitrarily two habitats of which the probability *p_transition_*(*n = 2*) = 1 − 89.28%. When one more habitat is added into the network, the probability of transitions among the three habitats can be calculated as *p_transition_*(*n = 3*) = *p_transition_*(*n = 2*) *×* (1 − 89.28%^2^), where the square of 89.28% represents the probability there is no direct transition between the added habitat and the former two habitats. Then we can expand such a procedure to estimate the probability for connecting *n* habitats in the transition network by

(5) $$p_{\mathrm{transition}}\left(n\right)=\{\begin{array}{c} 1-89.28\%\;,\;n=2\\ p_{\mathrm{transition}}(n-1)\times (1-89.28{\text{\%}}^{\left(n-1\right)}),\;n\;>\;2 \end{array}$$

### Transitive closure algorithm of microbiome network.

In the microbiome transition network, a closure is a subset of nodes (microbiomes) that are fully connected, so that each microbiome can be linked to any other sample by direct or indirect transitions (with finite transfer nodes). Closures can be initialized by an arbitrary node in the network, and then expanded by adding more external nodes that are directly connected with this closure (Fig. S6). If two or more closures are connected by any edge, these closures can also be merged as one closure. By the traversal among all nodes in the network *G* we get a main closure *C* with 98.31% of the samples.

10.1128/mSystems.00394-21.6 FIG S6 Expansion of a transitive closure by merging (A) linked node and (B) linked closure. Download FIG S6, TIF file, 0.4 MB. Copyright © 2021 Jing et al. 2021 Jing et al. https://creativecommons.org/licenses/by/4.0/ This content is distributed under the terms of the Creative Commons Attribution 4.0 International license.

### Size of the microbiome network.

In the main closure *C*, there are always multiple routes between two indirectly connected nodes (microbiomes). We count the edge between two directly linked nodes as 1, so the length of indirect routes is the number of transfer nodes + 1 on this route (Fig. S4). We used the *Dijkstra* algorithm (19) by Python package *igraph* (0.7.1 running inside Python 3.6.1) to find the pairwise shortest transition steps (with smallest number of transfer nodes) between all indirectly linked node pairs in the *main closure C*. Thus, the number of maximal steps among the shortest route is the diameter of the closure. The diameter means in this closure, any two microbiomes could be linked to each other by a route with steps that smaller than the diameter.

### Minimum spanning tree for the roadmap of microbiome network.

In a transitive closure, a spanning tree is a subnetwork that connects all nodes (microbiomes) with no cycle. For two directly linked samples *a* and *b*, we define their distance as

(6) dist(a, b)=1−sim(a, b)

the minimum spanning tree (MST) could be considered the global transition path of all samples with the highest overall transition probability, since it links all samples with the shortest total distance. In the main closure *C*, we used the Kruskal algorithm (23) to calculate the second-level MST to reflect the transition among different habitats from the global scale.

The first-level MST was on “sample resolution,” based on which we then made the second MST on “habitat resolution.” Initially we calculated the first-level MST of the main closure *C*, and then generated the habitat-based network *G′* (equation 2), where each node represents one habitat and the distance between two habitats *h_i_* and *h_j_* is the average distance of all edges that linked the two habitats in the MST. Then we computed the second-level MST (*G′*), which illustrates the global microbiome transition roadmap across multiple habitats.

$$G\prime =\left(V\prime =\left\{h_{i},h_{j},\ldots \;\right\},\;E\prime =\left\{\mathrm{average}\left(\mathrm{edge}\left(a,\;b\right)\right)\right\}\right)$$

(7) $$a\in h_{i},\;b\in h_{j},\;\mathrm{edge}\left(a,b\right)\in \mathrm{MST}\left(C\right)$$

The significance of the roadmap (MST[*G′*]) was assessed by the permutation test of the topologically equivalent subnetwork in the main closure *C* of the original network. Specifically, in a permutation, for each edge that connects two habitats (eg. *habitat_i_* and *habitat_j_*) in the roadmap, we also randomly selected an edge that connects two samples (eg. sample *a* and *b*), respectively, from these two habitats (eg. *a∈habitat_i_* and *b∈habitat_j_*). Since we iterated the permutation for 10,000 times, if the total distance of the roadmap is smaller than 99% of permutated network (also meaning the total probability is in the top 1%, *P* value < 0.01), we can consider the roadmap MST(G′) is significant in the main closure *C*.

### Search-based sample selection from reference database to link-separated closures.

To select transfer samples from a reference database to link two separated closures, we search all samples of each closure against the referenced repository for top matches with higher similarity than the direct transition cutoff (*T_d.t_* = 0.868), and the overlapped matches between the two closures are the transfer microbiomes that link the two closures. If there is no overlap in the matches, we then extend each of the closure by adding their matches and repeat the search process until we find any transfer sample. On the other hand, once closures cannot be further extended by database searching but still no available transfer sample is found, this means that no sample in the reference database is able to work as the transfer node to link the two separated closures by direct transition.

### Availability of data and materials.

The key bioinformatical tool here, Microbiome Search Engine (MSE), can be freely accessed as an online service via http://mse.ac.cn. Moreover, for standalone searches of customized microbiome databases, the kernel and tutorial of MSE are provided at GitHub (https://github.com/qibebt-bioinfo/meta-storms). All the data and analytical scripts used in this work are available at GitHub (https://github.com/qibebt-bioinfo/microbiomenetwork) to ensure reproducibility.
