# Supplementary figures and images for: A Scale-Free, Fully Connected Global Transition Network Underlies Known Microbiome Diversity
Source: mSystems. 2021 Jul 13;6(4):e00394-21. doi: 10.1128/mSystems.00394-21 (PMC8407412; doi:10.1128/mSystems.00394-21)

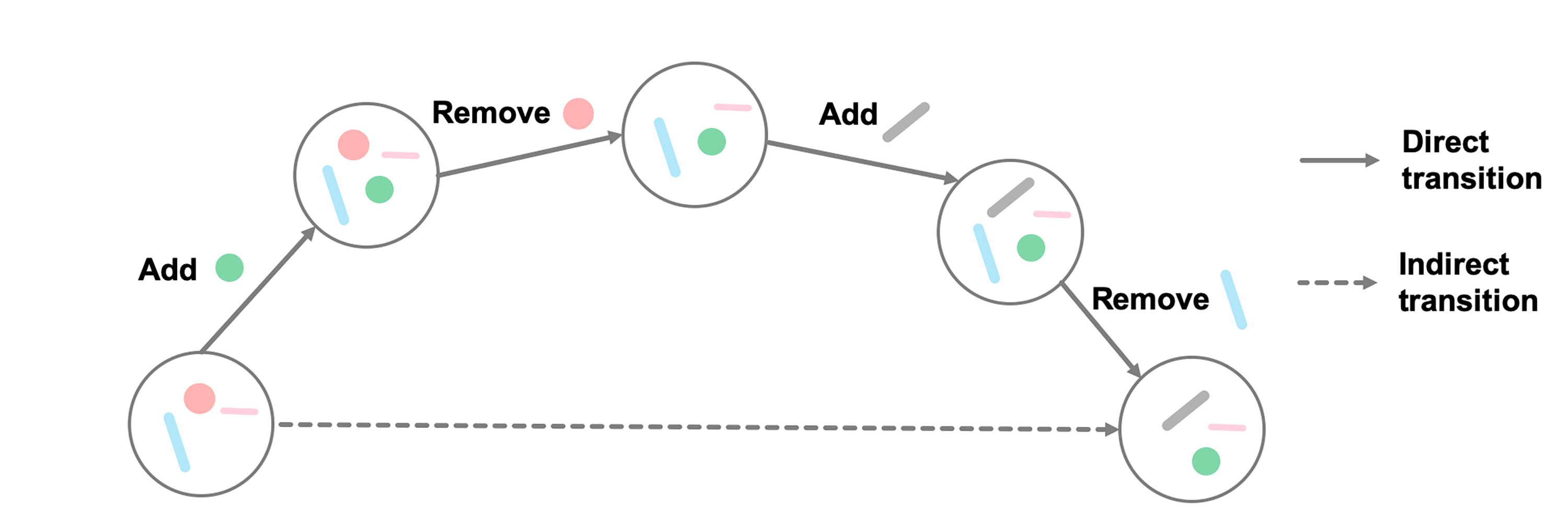

Supplement: FIG S1 [file msystems.00394-21-sf001.tif]

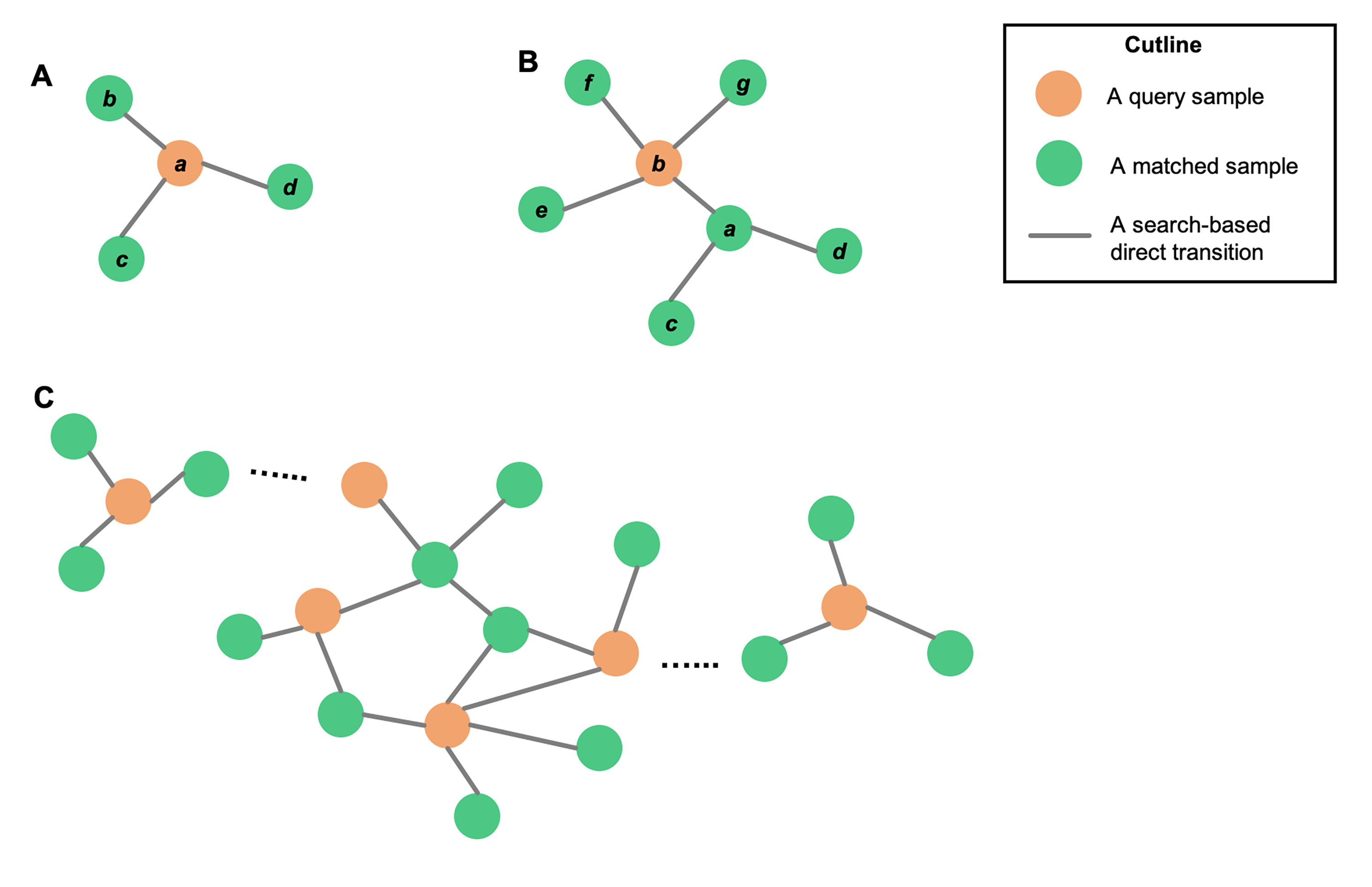

Supplement: FIG S2 [file msystems.00394-21-sf002.tif]

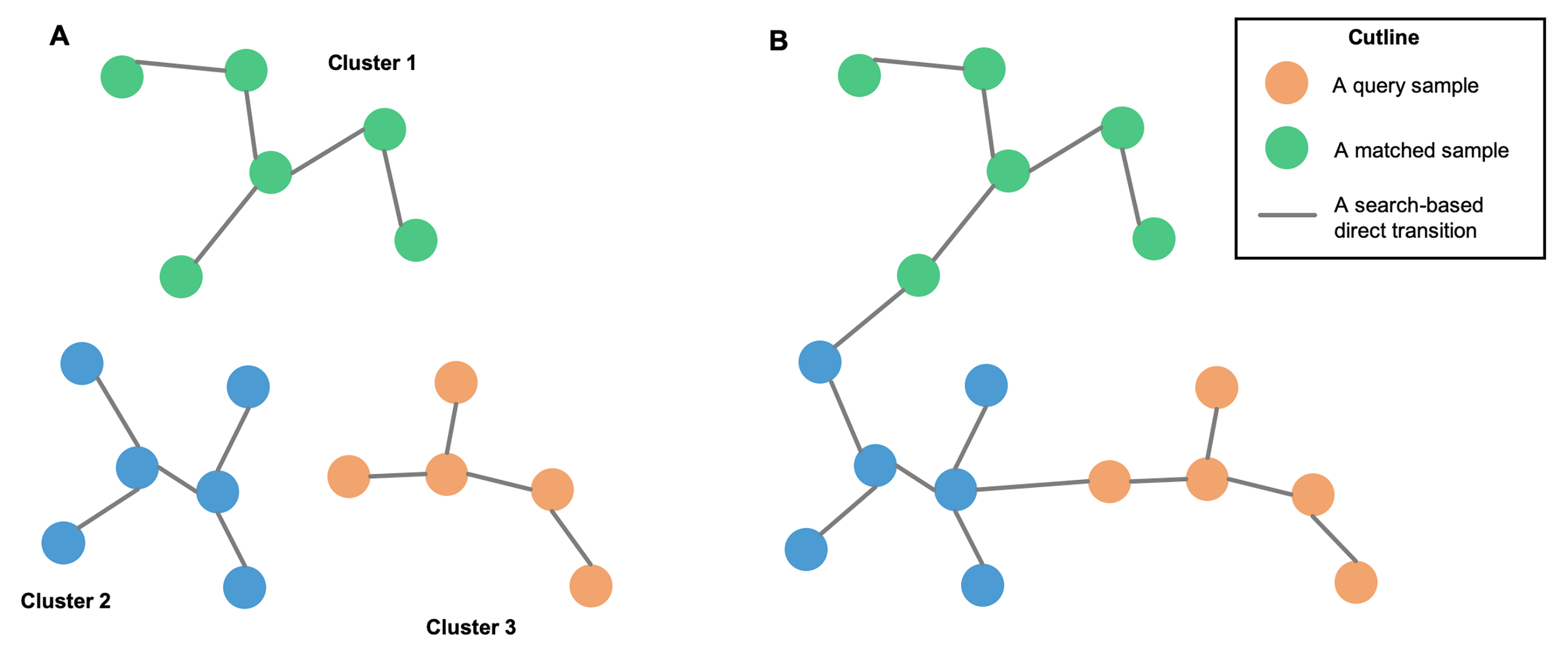

Supplement: FIG S3 [file msystems.00394-21-sf003.tif]

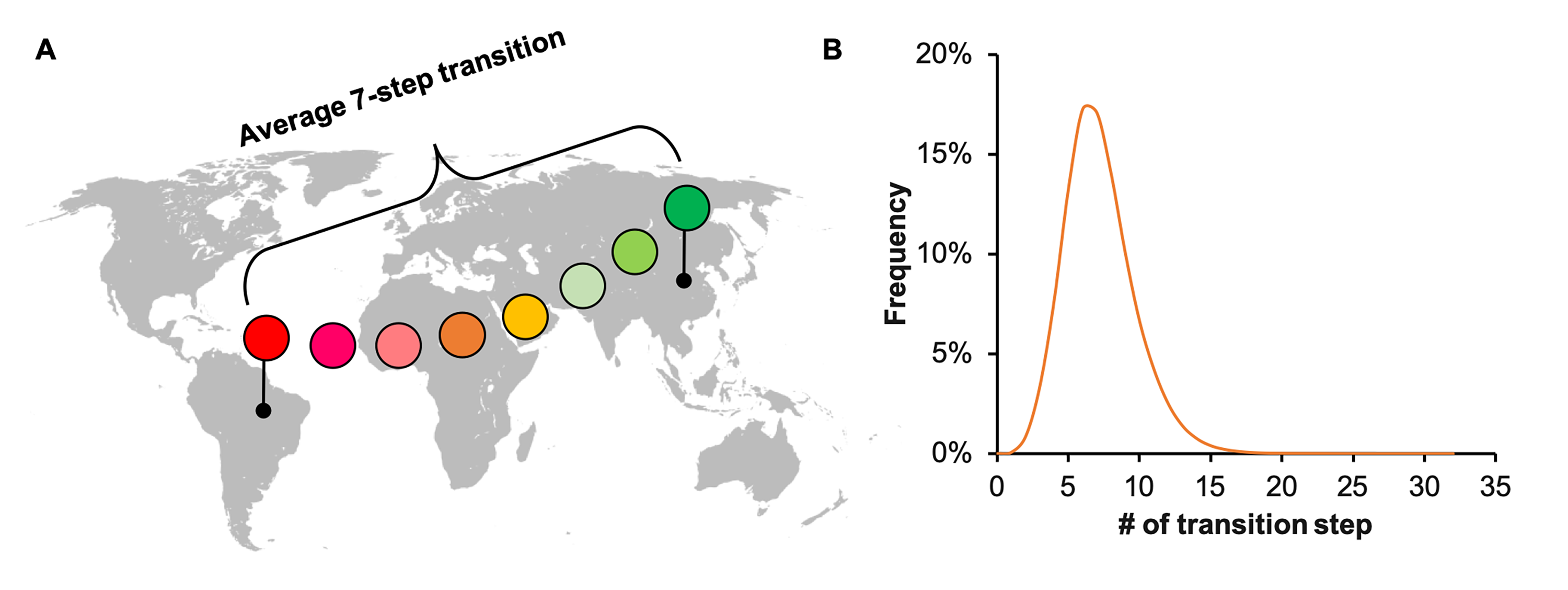

Supplement: FIG S4 [file msystems.00394-21-sf004.tif]

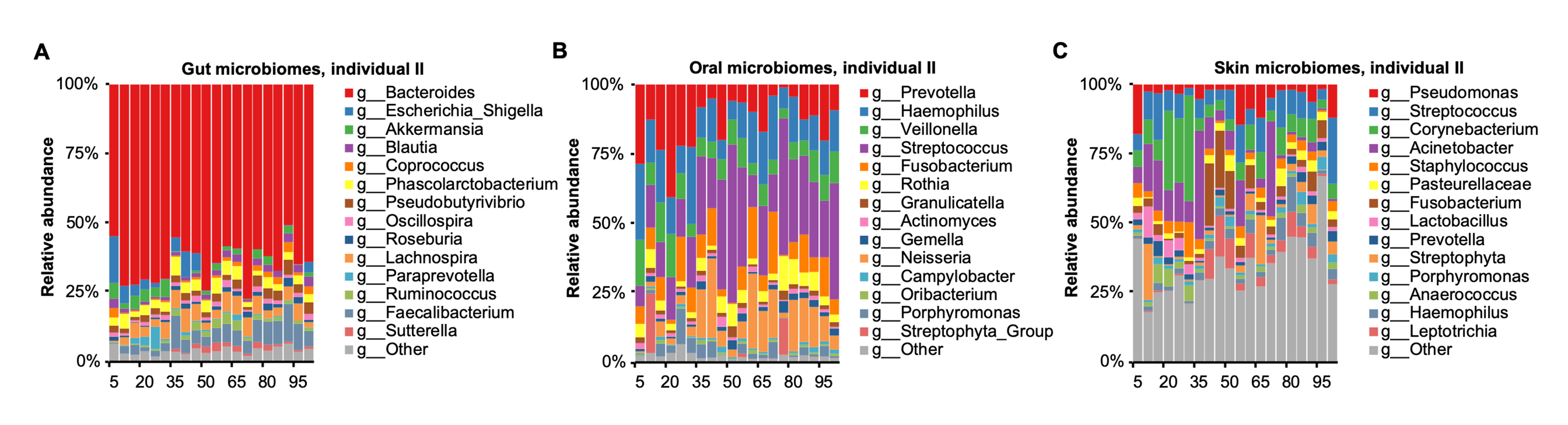

Supplement: FIG S5 [file msystems.00394-21-sf005.tif]

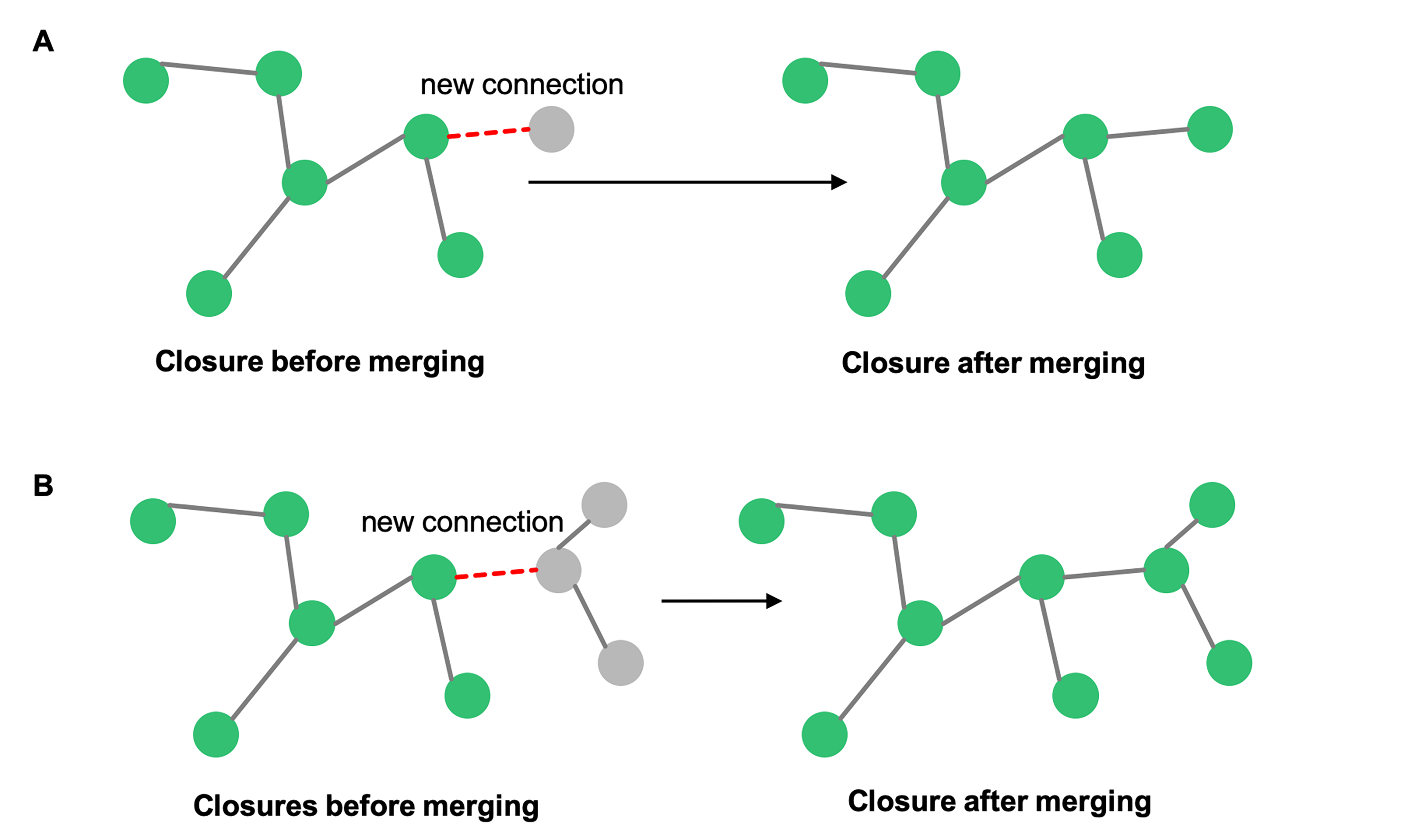

Supplement: FIG S6 [file msystems.00394-21-sf006.tif]
